# Pretreatment antigen-specific immunity and regulation - association with subsequent immune response to anti-tumor DNA vaccination

**DOI:** 10.1186/s40425-017-0260-3

**Published:** 2017-07-18

**Authors:** Laura E. Johnson, Brian M. Olson, Douglas G. McNeel

**Affiliations:** University of Wisconsin Carbone Cancer Center, 7007 Wisconsin Institutes for Medical Research, University of Wisconsin, Madison, 1111 Highland Avenue, Madison, WI 53705 USA

**Keywords:** Biomarker, DNA vaccine, Prostatic acid phosphatase, Prostate cancer, Interleukin 10

## Abstract

**Background:**

Immunotherapies have demonstrated clinical benefit for many types of cancers, however many patients do not respond, and treatment-related adverse effects can be severe. Hence many efforts are underway to identify treatment predictive biomarkers. We have reported the results of two phase I trials using a DNA vaccine encoding prostatic acid phosphatase (PAP) in patients with biochemically recurrent prostate cancer. In both trials, persistent PAP-specific Th1 immunity developed in some patients, and this was associated with favorable changes in serum PSA kinetics. In the current study, we sought to determine if measures of antigen-specific or antigen non-specific immunity were present prior to treatment, and associated with subsequent immune response, to identify possible predictive immune biomarkers.

**Methods:**

Patients who developed persistent PAP-specific, IFNγ-secreting immune responses were defined as immune “responders.” The frequency of peripheral T cell and B cell lymphocytes, natural killer cells, monocytes, dendritic cells, myeloid derived suppressor cells, and regulatory T cells were assessed by flow cytometry and clinical laboratory values. PAP-specific immune responses were evaluated by cytokine secretion in vitro, and by antigen-specific suppression of delayed-type hypersensitivity to a recall antigen in an in vivo SCID mouse model.

**Results:**

The frequency of peripheral blood cell types did not differ between the immune responder and non-responder groups. Non-responder patients tended to have higher PAP-specific IL-10 production pre-vaccination (*p* = 0.09). Responder patients had greater preexisting PAP-specific bystander regulatory responses that suppressed DTH to a recall antigen (*p* = 0.016).

**Conclusions:**

While our study population was small (*n* = 38), these results suggest that different measures of antigen-specific tolerance or regulation might help predict immunological outcome from DNA vaccination. These will be prospectively evaluated in an ongoing randomized, phase II trial.

**Electronic supplementary material:**

The online version of this article (doi:10.1186/s40425-017-0260-3) contains supplementary material, which is available to authorized users.

## Background

Over the last decade, immunological therapies have been investigated for the treatment of many types of carcinomas with FDA approvals of anti-CTLA-4 (ipilimumab) for melanoma, and anti-PD-1 or anti-PD-L1 (pembrolizumab, nivolumab, atezolizumab) for the treatment of melanoma, non-small cell lung cancer (NSCLC), renal cell carcinoma, head and neck cell carcinoma, bladder cancer, and Hodgkin lymphoma [1–9]. An anti-tumor vaccine, sipuleucel-T, was approved as a treatment for advanced, metastatic prostate cancer [10]. However, not all patients benefit from these therapies. When considering the cost burden and the potential to develop severe immune-related adverse events, at least with the checkpoint inhibitors, measures to predict individuals likely to respond, and measures to predict whether an individual is likely to develop autoimmune toxicity, are greatly needed. Similarly, early treatment biomarkers that could help identify responders at an early phase of treatment would be useful to know whether to continue a particular therapy that may exhibit delayed clinical benefit.

Recently, many efforts have been made to identify immunological markers that can be used to predict the efficacy of anti-tumor treatments for individual patients. Mainly investigated in studies involving T cell checkpoint inhibitor immunotherapies, tumor cell phenotype and the tumor microenvironment status such as infiltrating lymphocytes, localized T cell checkpoint receptor expression, and mutational burden have been reported as potential biomarkers for a number of malignancies [11–13]. Recently, pathological evaluation of tumors for the presence of immune-infiltrating cells has led to the identification of “hot,” “cold,” and “ignored” tumors that appear to be associated with the likelihood of response to immune checkpoint inhibitors [14]. Since obtaining tumor biopsies can be risky, expensive, incomplete (due to tumor heterogeneity) or infeasible (due to tumor location or absence of radiographically apparent disease) for many patients with advanced cancer, many have focused on peripheral blood markers, including serum proteins, cell-free nucleic acids, or immune cell subset populations [15–17].

In the case of prostate cancer, checkpoint inhibitors have demonstrated little benefit as monotherapies [18–21]. However, anti-tumor vaccines targeting specific proteins have demonstrated anti-tumor effect. Clinical trials targeting prostate tumor antigens such as prostatic acid phosphatase (PAP) and prostate specific antigen (PSA) have demonstrated the ability to elicit antigen-specific immune responses and have had a positive impact on the overall survival of patients with castration resistant metastatic prostate cancer (mCRPC) [10, 22–26]. For these antigen-specific therapies, the evaluation of antigen-specific immunity is feasible, and has been used as a biological marker of response to immunization. Moreover, the development of antibody and/or T cell responses to PAP after treatment with sipuleucel-T has been associated with greater overall survival [10, 27]. However, no markers to predict patients likely to respond to these therapies have been developed. This is particularly relevant to costly agents such as sipuleucel-T, or when considering combination therapies that might have greater toxicity. Preexisting antigen-specific T cells to prostate-specific antigens (PAP, PSA, and AR) have been reported in prostate cancer patients [28, 29]. The memory or regulatory phenotype of antigen-specific T cells has also been reported to affect the ability of a subject to develop a successful therapeutic immune response to antigen-targeted immunotherapies such as vaccines [30]. For example, Olson et al. reported the detection of PAP-specific effector responses after immunization with a DNA vaccine encoding PAP was inhibited by preexisting PAP-specific CD8 + CTL4 + IL-35-secreting regulatory T cells located in the peripheral blood of 30% of prostate cancer patients tested [30]. Additionally, De Gruijl et al. have demonstrated that prolonged overall survival following treatment with a cancer vaccine (GVAX) in combination with ipilimumab was observed in patients with advanced prostate cancer who had high pretreatment frequencies of CD4+ CTLA-4+, CD4+ PD-1+, or differentiated (non-naïve) CD8+ T cells, or alternatively low pre-treatment frequencies of regulatory T cells or differentiated CD4+ T cells [31]. Recently, a “peripheral immunoscore,” calculated by using previously reported functional-based immune cell subset criteria, was shown to predict overall survival benefit in both breast and prostate cancer patients that were receiving vaccine-mediated therapies along with conventional, nonimmune treatments [32]. Thus, obtaining an “immunoscore” by determining the key cell subsets (either positive or negative), or evaluating for the presence and phenotype of preexisting antigen-specific T cells, may be useful to identify which patients are likely to respond to immunotherapy treatments.

We have previously reported the results from two clinical trials using a DNA vaccine encoding prostatic acid phosphatase in patients with low-volume PSA-recurrent prostate cancer [33, 34]. In both trials some patients developed persistent Th1-biased immune responses specific for the target antigen, PAP, however many did not. The detection of persistent IFNγ-secreting T cell immunity as detected by ELISPOT was associated with favorable changes in PSA doubling time, suggesting that patients able to respond to immunization might have better outcomes [35]. While this is formally being tested in a blinded, randomized phase II clinical trial, these findings suggest that the identification of predictive biomarkers associated with long-term immune outcome could be beneficial to prioritize subjects most likely to benefit from anti-tumor vaccines. This is particularly relevant as immune therapies may take many months to demonstrate biological effect or disease stabilization, and which may not exhibit radiographic responses as early measures of clinical response, particularly for patients treated in the adjuvant or minimal residual disease settings who do not have radiographically identifiable disease. In the current report, we evaluated clinical laboratory parameters, the composition of peripheral blood cell immune populations, and measures of antigen-specific immunity present at baseline for association with subsequent development of immune response. Results of these studies will be employed in prospective randomized trials for validation.

## Methods

### Patient and sample populations

**Fig. 1:**
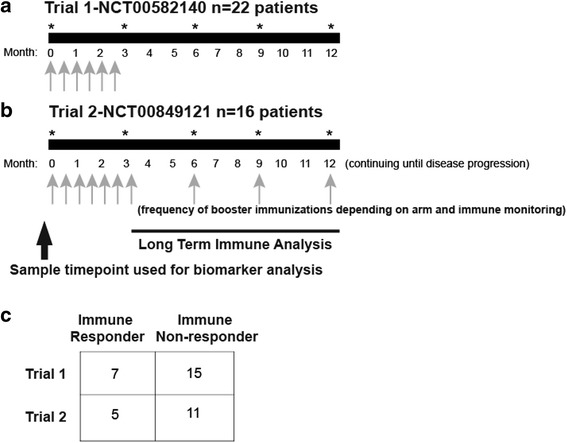
*Schema of vaccination schedule of two phase I trials in which prostate cancer patients were treated with a DNA vaccine encoding PAP*. Panel **a**: in Trial 1 (NCT00582140), patients received six biweekly immunization. Panel **b**: In Trial 2 (NCT00849121), patients received six biweekly immunizations followed by booster immunization every 3 months or based on immune monitoring. Grey arrows represent immunization time points. PAP-specific immune responses were monitored every 3 months after the initial six biweekly immunizations for each trial up to one year, as demonstrated by *asterisks*. Samples obtained before immunization were used for the biomarker analysis. Panel **c**: Shown are the number of immune responders and non-responders as defined for each trial

Peripheral blood mononuclear cells (PBMC), cryopreserved in liquid nitrogen and remaining from two clinical trials in which patients were treated with a DNA vaccine encoding prostatic acid phosphatase (PAP), were used for these studies. These trials enrolled patients with biochemically recurrent (rising PSA), non-metastatic prostate cancer that were either non-castrate (NCT00582140, *n* = 22) [33], or castration-resistant (NCT00849121, *n* = 16) [34]. Samples were collected under University of Wisconsin IRB-approved protocols, and all patients gave written, informed consent for remaining samples to be used for research. Trial schemes with schedules of vaccine adminstration and analysis timepoints are shown in Fig. 1. Samples or clinical laboratories from these subjects were classified as being from individuals who developed persistent immunity (immune responder, *n* = 12) or not (non-responder, *n* = 26). Immune responders were previously defined as those subjects who had PAP-specific IFNγ release detected by ELISPOT at least twice over a ≥ 3-month period in one year of follow up after the initial 12-week immunization series, with PAP-specific IFNγ release that was statistically significant compared with non-antigen-specific stimulation, at least 3-fold over the baseline, and with a frequency of at least 10 per 10^6^ PBMC [35].

### Immunophenotype analysis

Cryopreserved PBMCs were thawed, washed, rested for one hour at 37 °C, and filtered. To assess immune cell subtypes, PBMCs were stained with a panel of antibodies specific for CD4-PE-Cy 5.5 (clone SK3), CD33- PE (clone HIM3–4) (eBioscience, San Diego, CA), CD8-BV 605 (clone RPA-T8), CD14-Pac Blue (clone HCD14) (Biolegend, San Diego, CA), CD3-BV395 (clone UCHT1), CD56-PECy5 (clone B159), CD19-PECy7 (clone SJ258C1), HLA-DR PerCP Cy5 (clone G46–6), CD15-PE-CF594 (clone W6D3), CD66b-FITC (clone G10F5) (BD Biosciences, San Jose, CA), CD11c-APC (clone 3.9), and CD11b-APC Cy7 (clone M1/70) (Tonbo Biosciences, San Diego, CA) at 1:100 dilution for 30 min. at 4 °C. For the assessment of the T regulatory cells, PBMCs were stained for cell surface markers with antibodies specific for CD3-BV395, CD4-PE-Cy5.5, CD127-PE (clone HIL-7R-M21), and CD25-APC (clone 2A3) (BD Biosciences) at 4 °C for 30 min. Cells were then fixed using 2% formaldehyde (Polysciences, Inc. Warrington, PA), permeabilized (permeabilization buffer, eBioscience), and stained with an antibody specific for FoxP3-FITC (clone 206D) (Biolegend) for 30 min. at 4 °C. All samples were stained with a Live Dead marker, Ghost Dye V510 (Tonbo Biosciences) at 1:250 dilution and debris/dead cells were gated out of the analysis. Samples were analyzed on a BD Fortessa cytometer (BD Biosciences). Immunophenotype analysis was based on the flow cytometry gating strategies (Additional file 1: Figure S1) as standardized for the human immunology project [36]. MDSCs were defined as CD3-, HLA-DR^low/neg^, CD33+, CD11b+, CD14+ cells and Tregs were defined as CD3+, CD4+, CD127-, CD25+, FoxP3+ cells [37].

### Cytokine analysis

Cryopreserved PBMCs were thawed, washed twice with Hank’s balanced salt solution (Lonza, Walkersville, MD), and then rested for one hour at 37 °C. PBMCs were resuspended at 2 × 10^6^ cell/ml and cultured in T-cell medium (RPMI 1640 supplemented with L-glutamine, penicillin/streptomycin, ß-mercaptoethanol and 10% human AB serum) or Aim V medium (Invitrogen, Grand Island, NY) only (no antigen) or in the presence of 2 μg/ml PAP (Fitzgerald Industries, North Acton, MA), 2 μg/ml prostate specific antigen (PSA) (Lee Biosolutions, Maryland Heights, MO), 2 μg/ml androgen receptor ligand binding domain (AR LBD) (Invitrogen), or 5 μg/ml concanavalin A (Con A) (Sigma, St. Louis, MO) for 24–72 h at 5% CO_2_/37 °C. Supernatants were collected and analyzed for IFNγ, IL-2, IL-4, IL-6, IL-10, IL-17a, and granzyme B by cytokine bead array using standard methods (CBA flex kits, BD Biosciences). Samples were analyzed on an LSRII cytometer (BD Biosciences). Alternatively, for detection of antigen-specific IL-10 release, cryopreserved PBMCs were cultured in media only (no antigen), 2 μg/ml of a library of 15-mer peptides spanning the amino acid sequence of PAP or PSA and overlapping by 11 amino acids (LifeTein, Somerset, NJ), control peptide, or 40 ng/ml phorbol 12-myristate 13-acetate (PMA) and 1.3 μg/ml ionomycin for 5 h in the presence of 1.5 μM monensin for the last two hours. After incubation, cells were stained with CD4-PE-Cy5.5, CD3-BV395, CD8-BV605, CD19-PE (clone HIB19), CD56-PECy5, and Ghost dye780 (Tonbo Biosciences), permeabilized with cytofix/cytoperm (BD Bioscience), and stained with an antibody specific for IL-10-Alexa 488 (clone JES3-9D7, Biolegend). Samples were analyzed on an LSRII cytometer (BD Biosciences).

### *Trans Vivo* delayed-type hypersensitivity (DTH)

PBMCs (7.5–10 × 10^6^) were co-injected into the footpads of 6- to 8-week old SCID mice with phosphate-buffered saline (PBS, negative control), 25 μg tetanus/diphtheria toxoid (TT/D; Aventis Pasteur, Bridgewater, NJ) alone (positive control) or together with 1 μg of human PAP (Fitzgerald) or 1 μg of human PSA (Fitzgerald). Twenty-four hours later, the foot pad thickness was measured, in multiples of 10^−4^ in., using a dial thickness gauge (Mitutoyo, Kawasaki, Japan). The net antigen-specific swelling was measured as antigen-specific swelling subtracted for the contribution obtained with PBMC and PBS, as previously reported [30].

## Results

### Differences in absolute lymphocyte or monocyte counts in immune responding and non-responding patients were not detected

**Fig. 2:**
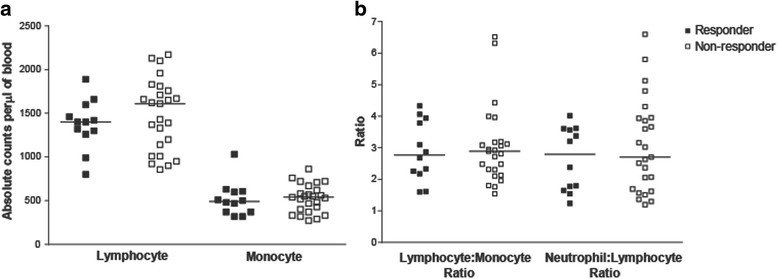
*Differences in absolute lymphocyte or monocyte counts in immune responding and non-responding patients were not detected*. Panel **a**: Absolute lymphocyte and monocyte counts per μl of blood of responder and non-responder patients. Panel **b**: The lymphocyte-to-monocyte and neutrophil-to-lymphocyte ratios were calculated. Each dot represents the absolute lymphocyte or monocyte counts and lymphocyte ratios from individual subjects (closed squares, immune responders (*n* = 12); open squares, non-responders (*n* = 25)). Lines show median values

**Fig. 3:**
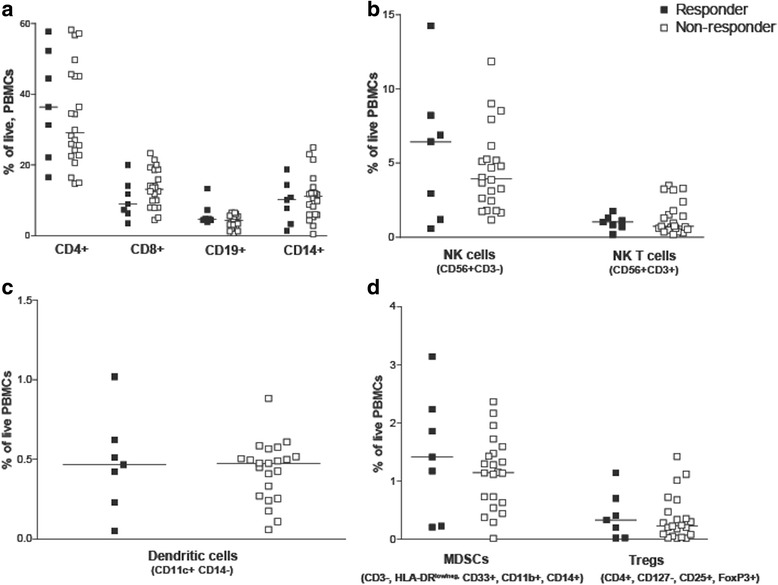
*Differences in peripheral blood cellular subsets were not detected in immune responding and non-responding patients*. The percentage of T cells (CD3 + CD4+ T cells or CD3 + CD8+ T cells), B cells (CD3-CD19+), monocytes (CD14+), (Panel **a**); NK cells (CD56 + CD3-), NK T cells (CD56 + CD3+), (Panel **b**); dendritic cells (HLADR + CD11c + CD14-), (Panel **c**); and MDSCs (CD3-HLA-DR^low/neg^CD33 + CD11b + CD14+) and T regulatory cells (CD3 + CD4 + CD127-CD25 + FoxP3+) (Panel **d**) was evaluated in PBMCs from immune responding and non-responding patients. Each dot represents the percentage of each cell type among total live PBMCs from an individual subject (closed squares, immune-responders (*n* = 7); open squares, non-responders (*n* = 22)). Lines represent median values for each data set. Samples were analyzed in duplicate and averaged. Cryopreserved samples were not available for this analysis for 9 subjects

Clinical laboratory data and peripheral blood samples were available from subjects treated in one of two clinical trials (Fig. 1). Patients were classified as immune responders or non-responders, as described above. Clinical laboratory data obtained within two weeks prior to beginning vaccination were evaluated for the number of lymphocytes, monocytes, neutrophils, and the ratio of lymphocytes-to-monocytes and lymphocytes-to-neutrophils. As shown in Fig. 2, no differences were observed in these parameters between immune responding and non-responding subjects. Similarly, peripheral blood samples obtained at baseline were evaluated for the frequency of different hematopoietic cell populations, including CD4+ and CD8+ lymphocytes, B cells, monocytes, natural killer cells, dendritic cells, CD4 + CD25 + FoxP3+ regulatory T cells, and myeloid derived suppressor cells (MDSC) (gating strategy demonstrated in Additional file 1: Figure S1). As shown in Fig. 3, the frequencies of these populations were not statistically different between immuneresponding and non-responding subjects.

### Antigen-specific immune responses exist in patients before vaccination

**Fig. 4:**
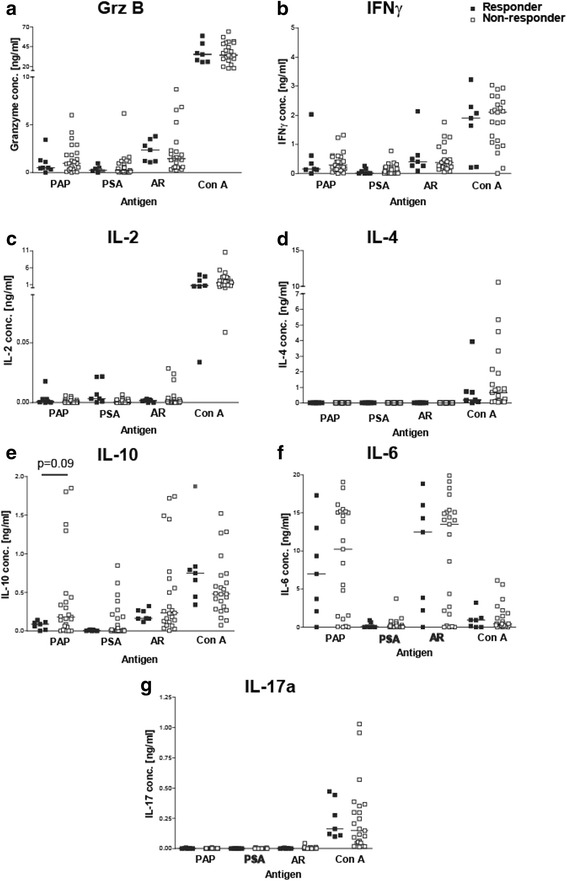
*Prostate cancer patients had preexisting immune responses to the PAP protein*. PBMCs from responder or non-responder patients were stimulated 1 or 3 day(s) with PAP, PSA, AR LBD (AR), and Con A. Supernatants from cultures were collected and analyzed for cytokine concentrations (**a**) Granzyme B, (**b**) IFNγ, (**c**) IL-2, (**d**) IL-4, (**e**) IL-10, (**f**) IL-6, and (**g**) IL-17a using a cytokine bead array. Each dot represents the cytokine expression level for an individual prostate cancer patient (closed squares-responders (*n* = 7) and open square-non-responders (*n* = 23)). Samples were analyzed in triplicates and averaged. Lines represent median values for each data set. Statistical comparisons were made using a Mann Whitney t test. As in Fig. 3, cryopreserved samples were not available for 8 subjects

We have previously reported that prostate antigen-specific immune responses exist in men with and without prostate cancer [28, 29]. We next investigated whether pretreatment PAP-specific immune responses were detectable in these subjects, and whether the type of T-cell immunity was associated with immune response outcome. PBMC were cultured in vitro with three different recombinant prostate-associated proteins, PAP, PSA, or the ligand-binding domain of the androgen receptor (AR LBD), and evaluated for antigen-specific cytokine secretion using a cytokine bead array analysis. Th1-type responses (IFNγ or granzyme B secreting), inflammatory-associated IL-6 responses, and IL-10-type regulatory responses were detected following stimulation with each of these prostate-associated proteins (Fig. 4). Th2-type responses (secreting IL-2 or IL-4) and Th17-type responses were not detected. Despite the presence of Th1 responses at baseline, PAP-specific secretion of IFNγ, granzyme B or IL-6 was not significantly different between immune responders and non-responders. However, PAP-specific secretion of IL-10 tended to be higher, albeit not significantly, in non-responding patients (Fig. 4e, p=0.09). In these patients, IL-10 was found to be expressed by both CD4+ and CD8+ T cells following similar analysis with flow cytometric intracellular cytokine staining (data not shown). Antigen-specific secretion of IFNγ, granzyme B, IL-6 and IL-10 were confirmed by ELISA using supernatants from antigen-stimulated T-cell cultures (data not shown). Next, we determined the number of patients with significant immune responses to one or more prostate cancer-associated antigens (compared to media alone). 88% patients had preexisting Th1 immune responses (IFNγ and Grz secreting) to the androgen receptor with the majority of these patients (52%) recognizing other prostate-specific antigens, PSA and PAP (Table 1). Interestingly, preexisting T cells of both Th1 and IL-10 secreting phenotypes were detected in the same patients. None of these patients had preexisting T cells that recognized PAP or PSA alone but only in combination with other prostate-specific antigens. There was no association between response to multiple antigens and development of long term Th1 immune response to the PAP vaccine target antigen (Table 1).

**Table 1:** *Patients had preexisting T cells to multiple prostate-specific proteins*. Shown are the number and % of patients with IFNγ-, granzyme B- (Grz), IL-10-, or IL-6-secreting responses to one or more (or none) of the three prostate cancer-associated proteins (PAP, PSA or AR)

	IFNγ	Grz	IL-10	IL-6
	n (%)	n (%)	n (%)	n (%)
None	4 (13.3)	3 (10)	1 (3.3)	9 (30)
PAP	0	0	0	0
PSA	0	0	0	0
AR	2 (6.6)	3 (10)	4 (13.3)	0
PAP + PSA	0	0	0	0
PSA + AR	0	1 (3.3)	0	0
PAP + AR	9 (30)	7 (23.3)	9 (30)	13 (43)
PAP + AR + PSA	15 (50)	16 (53.3)	16 (53.3)	8 (27)

### Antigen-specific regulatory immune responses exist in patients prior to vaccination

**Fig. 5:**
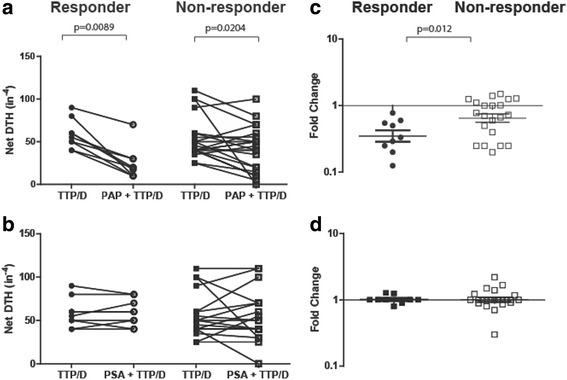
*Patients that were long term immune responders had preexisting PAP-specific regulatory responses*. Pretreatment PBMCs from immune responder (*n* = 9) and non-responder patients (*n* = 23) were injected into the footpads of SCID mice with TT/D (recall antigen) alone or in combination with PAP or PSA. Each dot represents the net swelling (10^−4^ in.) after 24 h. This was defined as the antigen-specific swelling measurement minus the swelling due to the PBMCs and PBS alone. Shown is the net DTH immune response (10^−4^ in.) to TT/D alone (closed circles, responders; closed squares, non-responders) or in combination with PAP (**a**) or PSA (**b**) (circles, immune responders; squares, immune non-responders) for each patient. Statistical comparisons were made using a paired, non-parametric t test analysis. The log-transformed fold change is shown comparing **c**) TT/D PAP to TT/D and **d**) TT/D PSA to TT/D for immune responders and non-responders. Comparison of fold change was made using a Mann Whitney t test

The detection of IL-10-secreting cells specific for PAP, higher in patients without evidence of subsequent Th1-biased immunity, suggested that the presence of antigen-specific regulation or tolerance might be important as a negative predictive factor to the development of immunity after vaccination. We have previously reported that a *trans vivo* delayed-type hypersensitivity (tvDTH) model, using peripheral blood cells and antigens placed into the footpads of SCID mice, could be used to evaluate different means of T cell immune regulation. We found that PAP-specific regulatory cells (CD8 + CTLA-4+), able to suppress a DTH response to a recall antigen, were detectable in some patients, with this regulatory effect mediated by IL-35 [30]. Using this method, we found that PAP-specific bystander immune suppression of a DTH response to a recall tetanus antigen was detectable in both responder and non-responder patients (Fig. 5a). However, the amplitude of suppression was significantly greater in immune responder patients (*p* = 0.012, Fig. 5c). In patients with PAP-specific IL-10 secreting T cells, DTH responses to PAP could not be detected if antibody to IL-10 was co-administered with PAP protein (data not shown). This suggests that preexisting PAP-specific T cells with bystander regulatory function are not associated with absolute tolerance, as individuals with this type of response had the capacity to respond to immunization. Moreover, this likely represents a different cell population than IL-10-secreting T cells, as was suggested by our prior study [30]. PSA-specific bystander immune suppression was only rarely detected, and was not associated with response to PAP-specific vaccination (Fig. 5b, d).

## Discussion

In this study we investigated different baseline immune measures, antigen-specific and antigen non-specific, as possible predictors of immunological response to antigen-specific vaccination using a DNA vaccine encoding the tumor antigen PAP. We found that non-antigen-specific immune measures, such as frequencies of circulating immune cell subsets, were not associated with the development of persistent Th1-biased T cell immune response to vaccination, whereas the presence and type of pre-existing regulatory-type antigen-specific T-cell immunity was most associated with the development of persistent IFNγ-secreting antigen-specific T cell immunity. These findings suggest that similar methods could be used to triage patients towards an ideal antigen-specific anti-tumor vaccine, and potentially that methods to block antigen-specific regulatory cells could improve the outcome from anti-tumor vaccines. Future studies will prospectively evaluate in validation studies whether the presence of antigen-specific IL-10-secreting immunity or bystander immune suppression can specifically serve as predictive immune biomarkers for antigen-specific vaccination.

There is currently great interest in the development of predictive and prognostic biomarkers for cancer immunotherapies [38, 39]. Much of this focus has been for T-cell checkpoint inhibitor therapies. The identification of predictive biomarkers is particularly relevant for immune-based therapies because not all patients derive immediate clinical benefit as determined by radiographic imaging, hence being able to identify patients unlikely to respond could save great cost and potential toxicity to these individuals. Higher baseline absolute leukocyte count (ALC) and relative lymphocyte count (RLC), and lower frequencies of circulating MDSC, have been identified as being associated with a favorable prognosis in melanoma patients treated with ipilimumab or pembrolizumb [15, 16]. Similarly, tumor biopsies demonstrating an “inflamed” phenotype with high numbers of tumor-infiltrating T cells, high expression of PD-L1 on tumor cells, and PD1 expression on infiltrating T cells have all been reported to be associated with clinical responses to pembrolizumab [3, 40–44]. In our study, tumor biopsy specimens were not available for analysis, a situation common to most trials and standard clinical practice, and therefore we focused on peripheral blood measures. A high circulating neutrophil to lymphocyte ratio (NLR) has been previously reported to be an adverse prognostic factor for patients with metastatic castration-resistant prostate cancer [45–47]. A high NLR ratio may indicate an increased neutrophil-dependent inflammatory response and decreased adaptive anti-tumor immune response. However we found that these non-specific peripheral blood immune measures, including pre-treatment lymphocyte subset and circulating MDSC frequencies, were not different between vaccine immune responders and non-responders.

We have previously reported that patients with prostate cancer can have existing Th1-biased immune responses specific for different tumor-associated antigens [28, 29]. We have also identified that PAP-specific, IL-35 expressing regulatory responses can be identified in patients [30]. In the current study, we specifically assessed whether different types of pre-existing immunity might affect the outcome of immunization in which the goal was to elicit a persistent Th1-biased immune response to the target antigen. Our results indicated PAP-specific secretion of IL-10 levels tended to be higher in immune non-responding patients, albeit not statistically significantly so. This implied that antigen-specific IL-10 secretion may limit the ability to generate a Th1-biased response with immunization. This is not surprising since IL-10 production has been associated with a tolerant phenotype. This further suggests that combining IL-10 blockade with immunization could be advantageous direction for future studies. In fact, several strategies are being investigated in preclinical studies to specifically block IL-10, including antibodies to IL-10 or its receptor, antisense oligonucleotides, and siRNA approaches, and an anti-IL-10 antibody is being investigated in human trials. It has been reported in a mouse melamona model that tumors engineered to overexpress IL-10 have less macrophage infiltration, lower expression of MHC class I molecules, and a more aggressive tumor phenotype, which could be abrogated by treatment with an anti-IL-10 antibody [48]. Moreover, Kalli et al. demonstrated in two different murine tumor models that combining a dendritic cell vaccine targeting gp100 along with anti-IL-10 treatment resulted in 100% tumor protection [49]. Such findings support the concept of combining antigen-specific vaccines with anti-IL-10 treatment in human trials.

Antigen-specific secretion of IL-10 tended to be observed in patients that did not develop persistent PAP-specific Th1 immunity following immunization, however our sample size was small and this was not statistically significant. Hence it will be important to test this prospectively in future trials using this specific DNA vaccine with a larger number of subjects. At present, it is unknown whether antigen-specific IL-10 secretion could serve as a general predictive biomarker for antigen-specific vaccines, or whether our findings are specific to this DNA vaccine. This is important, since sipuleucel-T is an FDA-approved vaccine for the treatment of advanced prostate cancer and similarly targets the PAP antigen [10]. To date, there are no predictive biomarkers for sipuleucel-T. If PAP-specific IL-10 secretion similarly identifies patients unlikely to immunologically respond to sipuleucel-T, that could save tremendous cost and time for individual patients. Moreover, our results suggest that some individuals develop antigen-specific IL-10 secretion to distinct tumor-associated antigens and not others, but overall that preexisting immune responses to two or more prostate cancer-associated proteins (AR, PAP, and/or PSA) were common (83% of individuals). This is particularly relevant for prostate cancer in which another anti-tumor vaccine, Prostvac, is in advanced stages of clinical testing [24]. If also approved, there could be two anti-tumor vaccines, targeting different prostate antigens, approved for the same patient population. Measures to identify patients more likely to respond to one therapy versus the other could be extremely useful to choose the best therapy for individual patients.

In this study, we observed that patients that were able to develop a long-term, IFNγ response after vaccination had pre-existing antigen-specific cells that elicited bystander suppression in a tvDTH model. This suggests the presence of PAP-specific, IL-35-secreting regulatory T cells in these patients, as we have previously reported [30]. This further suggests that the type of T cell regulation, as opposed to an IL-10-secreting and potentially tolerant response, may be predictive for response to antigen-specific immunization. Specifically, it is conceivable that the detection of this antigen-specific bystander regulatory phenotype indicates the presence of Th1-biased antigen-specific T cells that can be augmented with vaccination. Interestingly, our results from the DTH bystander suppressor assay suggest that these responses develop specific for some antigens but not others, as PAP-specific bystander suppression T cells were observed in more patients than PSA-specific bystander suppression. Clearly this is an area for future study, and specifically to determine whether PAP-specific IL-35 secretion might be a simpler means of detecting this type of antigen-specific regulation than using the in vivo SCID mouse DTH method. In fact, we did attempt to determine whether IL-35 could be measured directly by ELISA following antigen-specific stimulation in vitro*,* however we were unable to identify antibodies or commercial kits that could reliably detect IL-35 and distinguish it from other interleukins containing its IL-12p35 and Ebi3 subunits (data not shown).

## Conclusions

In summary, we sought to determine if measures of antigen-specific or antigen non-specific immunity were associated with immune response (the development of persistent antigen-specific Th1 immunity) to DNA immunization to identify possible predictive immune biomarkers. The frequency of different peripheral blood cell populations evaluated pretreatment was not associated with the the development of tumor antigen-specific Th1 cells following DNA immunization. However, non-immune responder patients tended to have higher antigen-specific IL-10 secretion pre-vaccination, suggesting this might be investigated as a negative predictive biomarker for immune response to PAP-targeted DNA immunization, at least using a vaccine as a monotherapy. Our results also suggest that the identification of antigen-specific bystander suppression, detected in our study using a *trans-vivo* DTH assay in SCID mice, might be investigated as a positive predictive biomarker for immune response to immunization. Our study was limited by a small sample size (*n* = 38), multiple comparisons with the overall small sample set, and the retrospective design of this analysis. Consequently, we plan to prospectively test these findings in an ongoing randomized phase II trial evaluating this same DNA vaccine in patients with early, recurrent prostate cancer (NCT0134652).

## Additional file

Additional file 1: Figure S1.
*Flow cytometry gating strategy for the immunophenotype analysis*. Dead cells were excluded by gating on the negative cell population for the live dead marker, Ghost Dye V510. Duplicates were removed by progressive gating on FSC-A and FSC-H. A morphological gate was defined using SSC-A and FSC-H. A) The gating strategy for populations of monocyte, dendritic cells, and natural killer cells were defined as the following markers: monocyte (CD3-CD19-CD14+), dendritic cells (CD3-CD19-HLADR+CD11c+), and natural killer cells (CD3-CD19-CD56+) [37]. MDSC cells were defined as Lin- (CD3-CD19-), HLADRlow, CD33+CD11b+. B) The gating strategy for populations of B cells and T cells were defined as the following: CD8+ T cells (CD3+ CD8+), CD4+ T cells (CD3+CD4+), and B cell lymphocytes (CD3-CD19+). Regulatory T cells were defined as CD4+CD127^low^CD25+FoxP3+. CD25+ and FoxP3+ gating were based on FMOs. (DOCX 467 kb)

## Abbreviations

AR: androgen receptor
CTLA-4: cytotoxic T lymphocyte antigen-4
DNA: deoxyribonucleic acid
DTH: delayed type hypersensitivity
ELISPOT: enzyme-linked immunosorbent spot assay
IL-X: interleukin-X
MDSC: myeloid derived suppressor cell
PAP: prostatic acid phosphatase
PBMC: peripheral blood mononuclear cells
PSA: prostate specific antigen
