# Supplementary material for: Pretreatment antigen-specific immunity and regulation - association with subsequent immune response to anti-tumor DNA vaccination
Source: J Immunother Cancer. 2017 Jul 18;5:56. doi: 10.1186/s40425-017-0260-3 (PMC5514519; doi:10.1186/s40425-017-0260-3)
Supplement: Additional file 1: Figure S1. — Flow cytometry gating strategy for the immunophenotype analysis. Dead cells were excluded by gating on the negative cell population for the live dead marker, Ghost Dye V510. Duplicates were removed by progressive gating on FSC-A and FSC-H. A morphological gate was defined using SSC-A and FSC-H. A) The gating strategy for populations of monocyte, dendritic cells, and natural killer cells were defined as the following markers: monocyte (CD3-CD19-CD14+), dendritic cells (CD3-CD19-HLADR+CD11c+), and natural killer cells (CD3-CD19-CD56+) [37]. MDSC cells were defined as Lin- (CD3-CD19-), HLADRlow, CD33+CD11b+. B) The gating strategy for populations of B cells and T cells were defined as the following: CD8+ T cells (CD3+ CD8+), CD4+ T cells (CD3+CD4+), and B cell lymphocytes (CD3-CD19+). Regulatory T cells were defined as CD4+CD127lowCD25+FoxP3+. CD25+ and FoxP3+ gating were based on FMOs. (DOCX 467 kb) [file 40425_2017_260_MOESM1_ESM.docx]

**Additional file 1: Figure S1**
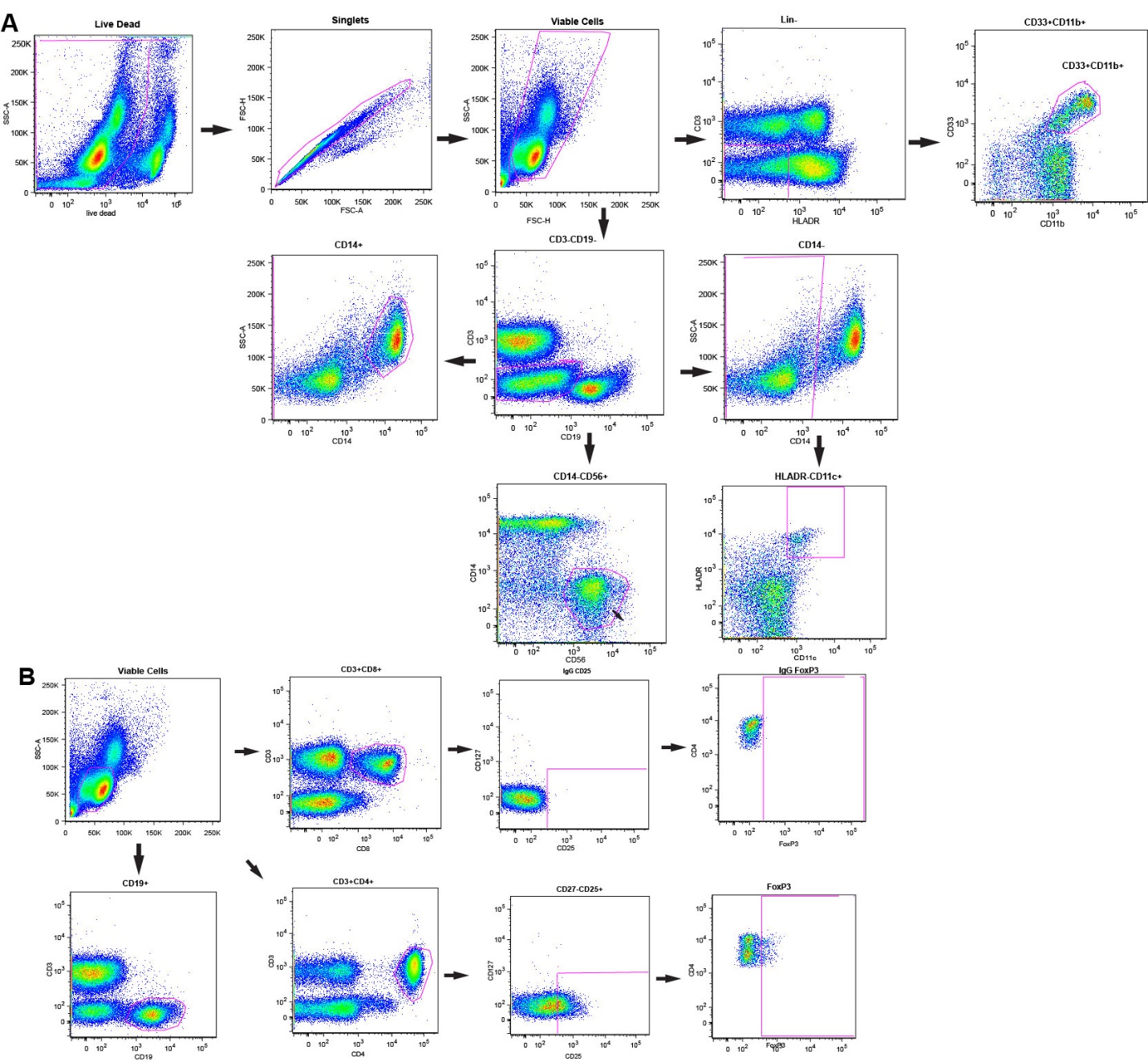


**Supplementary Figure 1**: *Flow cytometry gating strategy for the immunophenotype analysis*. Dead cells were excluded by gating on the negative cell population for the live dead marker, Ghost Dye V510. Duplicates were removed by progressive gating on FSC-A and FSC-H. A morphological gate was defined using SSC-A and FSC-H. A) The gating strategy for populations of monocyte, dendritic cells, and natural killer cells were defined as the following markers: monocyte (CD3-CD19-CD14+), dendritic cells (CD3-CD19-HLADR+CD11c+), and natural killer cells (CD3-CD19-CD56+) [[37](#_ENREF_37)]. MDSC cells were defined as Lin- (CD3-CD19-), HLADRlow, CD33+CD11b+. B) The gating strategy for populations of B cells and T cells were defined as the following: CD8+ T cells (CD3+ CD8+), CD4+ T cells (CD3+CD4+), and B cell lymphocytes (CD3-CD19+). Regulatory T cells were defined as CD4+CD127^low^CD25+FoxP3+. CD25+ and FoxP3+ gating were based on FMOs [[51](#_ENREF_51)].
